# Bridging Technology and Nutrition: A Systematic Review of AI and XR Applications for Nutritional Insights in Restaurants and Foodservice Operations

**DOI:** 10.3390/nu18091364

**Published:** 2026-04-25

**Authors:** Younes Bordbar, Jinyang Deng, Brian King, Hyunjung Lee, Wenjia Zhang

**Affiliations:** 1 Arch H. Aplin III ’80 Department of Hospitality, Hotel Management and Tourism, Texas A&M University, College Station, TX 77843, USA; younes.bordbar@tamu.edu (Y.B.); brian.king@ag.tamu.edu (B.K.); ttzhwj1999@tamu.edu (W.Z.); 2 Department of Nutrition, Texas A&M University, College Station, TX 77843, USA; grace.lee@ag.tamu.edu

**Keywords:** menu to meal, artificial intelligence, extended reality, nutritional labeling, restaurant, foodservice operations

## Abstract

**Purpose**: This study provides a critical examination of the literature on applying artificial intelligence (AI) and Extended Reality (XR) in restaurant settings and related foodservice operations. It focuses on how AI and XE influence consumer nutrition awareness and decision-making about food choices, and their implications for customer satisfaction, loyalty, and service delivery in foodservice environments. **Design**/**methodology**/**approach**: The study adopts a systematic literature review (SLR) approach following the PRISMA method. An initial search identified over 3900 academic papers published between 2016 and 2025. Studies were selected on the basis of predetermined inclusion and exclusion criteria, and 26 peer-reviewed articles were analyzed. The review provides a conceptual synthesis and develops propositions for practical applications and future research directions. **Findings**: The review reveals a shift from static systems that rely on optimization, toward adaptive and user-centered solutions that are behavior-oriented. AI applications predominate in the case of calorie tracking, personalized recommendations, and menu planning. Though deployment of XR technologies (e.g., AR and VR) is less prevalent, they offer potential for immersive, and real-time interventions. A key distinction emerges between studies demonstrating empirical effectiveness (e.g., improved understanding and healthier choices) and those focused on technical and/or conceptual developments. To date, there has been limited validation of behavioral impacts in foodservice settings. **Originality**: This study offers a theory-informed conceptualization of AI and XR applications in restaurant and foodservice contexts by integrating three perspectives: hospitality (menus and dining experience), nutrition (dietary awareness and healthier choices), and human–technology interaction (technology acceptance and user engagement). The study reconceptualizes AI- and XR-enabled systems as behavioral intervention tools and outlines a focused research agenda for advancing nutritional communication in foodservice environments.

## 1. Introduction

Artificial Intelligence (AI) and Extended Reality (XR) have become prominent contemporary phenomena [1], reshaping the way individuals and organizations interact with their environments [2]. Such technologies not only enhance awareness [3] but also provide innovative tools for decision-making [4], personalization [5], and efficiency [6]. The food and beverage (F&B) sector plays a particularly significant role within the hospitality and tourism domain [7], through its close association with consumer experience, service quality, and customer satisfaction [8].

While AI and XR technologies have advanced across various sectors of the economy, their integration within the restaurant industry has been limited, notably in the domain of menu design. Despite their potential to enhance consumer decision-making, improve menu personalization, and promote healthier eating behaviors, these technologies have not yet become commonplace in foodservice operations. Restaurants are still characterized by traditional methods of menu presentation and dietary information [9], with limited implementation of AI-powered systems and XR applications, such as AR menus or interactive nutritional recommendations. This adoption lag underscores the importance of exploring how these technologies could be leveraged in foodservice to improve nutrition awareness and guide healthier consumer choices.

The significance of this research is underscored by the growing demand for health-conscious dining options [10] and the increasing prevalence of chronic diseases that are linked to poor dietary habits [2]. AI and XR have the potential to revolutionize how nutritional information is presented in restaurants and foodservice operations and to engage consumers more actively in healthier food choices. By bridging the gap, this study will enlighten the current state of AI and XR in foodservice, identify the opportunities and challenges for adoption, and explore the theoretical underpinnings that guide acceptance by diners of these technologies.

Despite the growing body of research on artificial intelligence, extended reality, and digital health applications, existing scholarship remains fragmented across technical, clinical, and consumer-oriented domains. Prior studies have primarily focused on technical and algorithmic nutrition recommendation systems, automated dietary monitoring and food sensing technologies, and technology-driven food design and sensory innovations, rather than integrated AI and XR interventions within restaurant foodservice environments. However, to our knowledge, no systematic review to date has holistically integrated AI and XR applications within restaurant and on-site foodservice environments through a behavioral and hospitality-focused theoretical lens. As a result, the field lacks a unified conceptual understanding of how these technologies function not merely as informational tools, but as behavioral intervention mechanisms capable of shaping nutrition awareness and food choice decisions at the point of consumption.

To address this gap, the present study advances hospitality technology research in three important ways. First, it reconceptualizes AI- and XR-enabled menu systems as behavioral health interventions rather than purely operational or algorithmic systems. Second, it integrates the Technology Acceptance Model (TAM) [11] and the Health Belief Model (HBM) [12] into a unified framework that explains how technological characteristics translate into nutrition awareness and ultimately health-conscious food choices in restaurant settings. Third, it identifies an evolutionary shift in the literature from static optimization models toward immersive, adaptive, and persuasive AI ecosystems that blend personalization, explainability, and experiential engagement. By bridging hospitality management, nutrition science, and human–technology interaction, this study offers a theoretically grounded agenda for the next generation of intelligent foodservice systems.

In addressing the identified gaps, this study pursues the following objectives. First, it systematically reviews the existing literature on the application of AI and XR in restaurant and foodservice settings, with a particular focus on nutritional labeling and dietary decision-making. Second, it synthesizes the key technological applications and proceeds to categorize them, based on their functional roles in supporting nutrition awareness and food choice behavior. Third, it develops a conceptual framework integrating the TAM and HBM to explain how AI and XR technologies influence consumer awareness and health-conscious decisions. Finally, the study proposes a research agenda that identifies key gaps and directions for future researchers in AI- and XR-enabled foodservice environments.

## 2. Literature Review

Consistent with the study objectives, this section reviews prior research on AI- and XR-enabled technologies across three domains: (1) nutrition and dietary monitoring, (2) food recommendation and menu systems, and (3) immersive and interactive dining technologies. The review aims to position existing studies within a unified conceptual perspective and to highlight the fragmented nature of current research across technical, clinical, and hospitality-focused contexts.

Numerous studies have demonstrated that food intake and eating habits have a significant influence on the health of individuals [13] and on overall life satisfaction [2]. Restaurants and other foodservice settings are therefore expected to play a critical role in supporting these outcomes. One important avenue for achieving this is through menu design. Nie et al. [14] conducted a systematic review about menu design and concluded that menus extend beyond a listing of dishes. They are influenced by factors such as social norms, cultural values, market forces, industry practices, and customer preferences. Menus function as important tools for communication and management, helping restaurants to connect with guests and guide their dining decisions.

Recognizing the importance of food intake and eating habits on people’s health, Hassannejad et al. [15], conducted a literature review to categorize research on automating the monitoring of diets. They proposed two study classifications: one focused on extracting information about dietary content from images, and the other on using wearable sensors to detect eating behaviors. In another investigation, Bo et al. [16] explored the field of food design, highlighting how AI and 3D printing technologies are influencing the industry and helping restaurants achieve their goals, such as conveying messaging from the chef to customers through innovative culinary presentations. Collectively, these studies illustrate how Artificial intelligence (AI) Artificial intelligence (AI) has the capacity to transform raw dietary data into actionable insights, thereby offering the potential to support personalized and real-time dietary guidance.

AI can play a significant role in achieving the previously noted goals. Various subfields of AI have been applied for this purpose, including, but not limited to, machine learning (ML) [17], computer vision (CV) [18], natural language processing (NLP) [19], recommender systems [10], deep learning [20], reinforcement learning [21], Knowledge-Based Systems [22] and data mining [23]. Collectively, these technologies enable applications ranging from personalized meal recommendations and calorie tracking to menu optimization and predictive modeling of consumer choices, demonstrating AI’s multifaceted role in enhancing nutritional awareness [10,17,23].

An overview article [24] examined recommendation techniques, within the healthy food context and tailored to both individuals and groups. They also assessed current advances in food recommender systems and outlined key challenges for the progression of future recommendation technologies in this field. Another systematic literature review on nutrition recommendation systems (NRS) was conducted by Abhari et al. [25], who reported that hybrid recommender systems (40%) and knowledge-based recommender systems (32%) were the most employed types within this domain. Saad and Islam [26] also conducted a scoping review on real-time food nutrition classification and recommendation systems. They found that machine learning algorithms and sensor-based technologies are used to support smart dietary decisions, highlighting AI’s direct role in analyzing dietary data, predicting user needs, and delivering personalized nutritional guidance.

XR is another technology applied to support these outcomes, encompassing several key subfields such as virtual reality (VR) [27], augmented reality (AR) [28], and mixed reality (MR) [29]. These technologies enable immersive dining experiences [30], interactive menu visualizations [31], and enhanced customer engagement [32], helping restaurants communicate culinary concepts [33], educate consumers about nutritional content [34], and guide food choices [35] in innovative ways. XR thus complements AI by providing experiential and visual interventions that reinforce algorithmic nutritional information, linking cognitive understanding with experiential engagement in food choice contexts.

## 3. Methodology

A systematic review approach was employed to achieve a holistic understanding of using AI and XR trends in restaurants and other foodservice operations related to nutritional labeling and informing. The review process adhered to the PRISMA guidelines [36], ensuring transparency, rigor, and a systematic approach to the identification, selection, and analysis of relevant studies. An extensive search was conducted across nine electronic databases, including Scopus, ScienceDirect, Web of Science, Emerald Insight, PubMed, ACM Digital Library, IEEE Xplore, Hospitality & Tourism Complete, and APA PsycINFO, in June 2025 to identify relevant studies. These databases were selected to ensure comprehensive and multidisciplinary coverage of the literature, encompassing hospitality, nutrition, health, and technology-related research domains. In addition to the major indexing databases, subject-specific databases were included to improve coverage and reduce the risk of omitting relevant studies not indexed in broader platforms.

Seven out of nine databases were searched using the following terms: [(menu OR meal OR restaurant OR foodservice) AND (“artificial intelligence” OR “augmented reality” OR “virtual reality”) AND (“nutritional labeling” OR nutrition)]. Search strategies were adapted to acknowledge the differences in search syntax and limitations between databases. For seven of the databases, the search string was used as written. However, for Web of Science and IEEE Xplore, the search strategy was modified to accommodate database-specific search structures, with the combined query decomposed into all possible keyword combinations (4 × 3 × 2 = 24) and conducted separately (e.g., [menu AND “artificial intelligence” AND “nutritional labeling”]). Quotation marks (“ ”) were also used to ensure that multi-word terms, such as “augmented reality” were retrieved as exact phrases. This review included studies from the past decade (2016–2025) to ensure that the findings reflect the most recent trends and developments in the field.

The selection of search terms was guided by the study’s specific focus on the intersection of (1) restaurants and foodservice contexts, (2) AI and XR technologies, and (3) nutritional labeling and nutrition-related information at the point of decision-making. The selected keywords were narrowed purposely to capture studies that address menu-related and on-site foodservice environments explicitly, rather than broader dietary or health domains. Closely related terms were also considered during the development of the search strategy though were ultimately excluded to maintain conceptual precision and avoid scope dilution. The final keyword string was designed intentionally to balance relevance and coverage while remaining aligned with the review’s specific focus. Studies were identified based on predetermined eligibility criteria, as summarized in Table 1.

Studies were included if they were peer-reviewed empirical articles published between 2016 and 2025, written in English, and examined the application of artificial intelligence or extended reality technologies in restaurants or foodservice settings, particularly in relation to menus and nutritional labeling. These criteria were defined to ensure the inclusion of recent, high-quality research directly relevant to consumer-facing nutritional information in foodservice contexts. Studies were excluded if they were not available in full text despite reasonable access efforts, were non-peer-reviewed or review-based, or fell outside the foodservice context (e.g., personal dietary tracking). Research conducted in healthcare settings, such as hospitals or clinics, was also excluded, as clinical nutrition applications were beyond the scope of this review.

**Figure 1:**
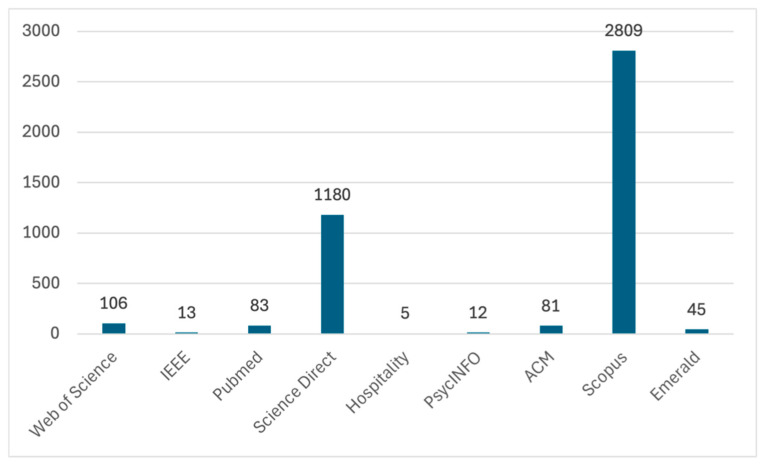
Number of records by database.

The selection of studies was conducted using Covidence, a web-based tool designed to streamline the screening of titles and abstracts in systematic reviews [37]. An initial total of 4,337 records was identified through database searches, with 3929 unique records remaining following the removal of duplicates. Gray literature sources such as conference papers were excluded by filtering databases such as Hospitality & Tourism Complete and APA PsycINFO to include only peer-reviewed articles. Gray literature records may have been retrieved in the case of databases without a peer-review filter. However, any articles that met the inclusion criteria during the screening process were checked carefully to ensure that gray literature was excluded from the final analysis. The distribution of records retrieved from each database is presented in Figure 1. Title and abstract screening were performed independently by two authors following the inclusion criteria. Each article was categorized as ‘yes’ (eligible), ‘maybe’ (potentially eligible), or ‘no’ (ineligible). Consensus was reached for 3527 articles, corresponding to an initial inter-rater agreement rate of 89.8%, which exceeds the commonly accepted minimum threshold of 80% for adequate agreement in research [38]. Disagreements regarding the remaining articles were addressed through discussion between the reviewers, leading to the selection of 204 articles for full-text assessment.

**Figure 2:**
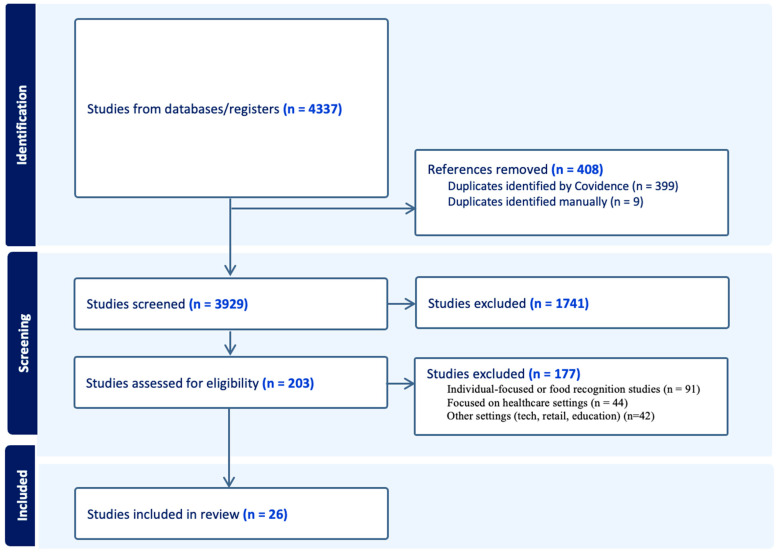
PRISMA flow diagram.

Of the 204 full-text articles assessed for eligibility, 91 were excluded because of their focus on individual dietary needs rather than restaurants and foodservice contexts, or because they developed deep learning-based food recognition and dietary assessment systems for analyzing daily meal images. An additional 44 articles were excluded because they primarily addressed healthcare settings, and the remaining 42 articles were excluded for other reasons, such as focusing on health tech companies, smart kitchens, supermarkets, or education, resulting in 26 articles being included in the review. Of the initial 3929 records, only 26 studies were included because the review focused specifically on restaurant and foodservice contexts, applied strict inclusion/exclusion criteria, and considered only primary empirical studies, ensuring that the selected studies provide directly relevant and high-quality evidence. The procedure for selecting articles is illustrated in the PRISMA flow diagram (Figure 2). A structured data extraction form was utilized to collect the relevant information, which included sections for:

- Study details (authors, publication year, and source journal).
- Targeted foodservice in the study
- Mapped AI/XR category; and
- Key contribution/finding

Following full-text selection, the extracted data were synthesized using a narrative synthesis approach supported by inductive thematic analysis. This approach was adopted to integrate heterogeneous studies drawn from hospitality, nutrition, and computer science. The synthesis focused on identifying recurring technological applications, research objectives, and outcomes related to nutritional information and consumer decision-making in foodservice settings. A structured data extraction matrix was used to code each study according to predefined fields, including foodservice context, AI/XR technology type, and key contributions. Coding was conducted iteratively, with studies repeatedly reviewed and compared to identify recurring patterns and conceptual similarities.

Thematically related studies were grouped through this iterative comparison process, leading to the identification of five main research categories: Thematically related studies were grouped through this iterative comparison process, leading to the identification of five main research categories: (1) AI-powered calorie and nutrition tracking, focusing on automated dietary assessment and intake monitoring; (2) AI-driven personalized food recommendations and meal guidance, emphasizing adaptive and health-oriented recommendation systems; (3) AI-driven menu planning, addressing optimization of menus based on nutritional, cost, and operational factors; (4) AI-based promotion of healthier eating choices, incorporating behavioral nudges, explainability, and persuasive system design; and (5) immersive and real-time dietary interventions, highlighting the use of XR technologies such as AR and VR to influence food perception and decision-making. The coding framework was then constantly refined to ensure consistency, conceptual clarity, and align with the review objectives.

In addition to the five primary research categories, the review also identified three additional areas that, while relevant, were too broad to be addressed systematically within this study. These emerging categories include personalized meal plans for individual consumers, food recognition and dietary assessment systems (technology-driven focus), and AI/XR applications in healthcare settings for nutritional management. Each of these domains warrants separate, focused systematic reviews due to the depth and specificity of the research.

To ensure the reliability and rigor of this systematic literature review, all included studies were evaluated using the quality appraisal checklist [39], which assesses quality across nine domains, including title and abstract, introduction and aims, method and data, sampling, data analysis, results, implications, transferability, and ethical considerations. Each study was scored on a scale from 1 (very poor) to 4 (good) for each domain, with a total possible score of 36 points. Scores between 28 and 36 indicate high-quality studies, 20 to 27 suggest fair quality, 10 to 20 reflect poor quality, and below 10 is considered very poor quality. The 26 included studies received quality scores ranging from 26 to 36 points (mean = 31.7, SD = 2.1). Of the various studies, 25 were rated as high quality and just 1 as fair. Across thematic categories, Personalized Food Recommendation studies generally scored higher (mean = 32.7), whereas AI-Driven Menu Planning had slightly lower scores (mean = 30.5). Additionally, 22 of the included articles were published in Q1 journals and 4 in Q2 journals, further demonstrating the high academic quality of the studies analyzed. A summary of the quality ratings is presented in Table S1, providing an overview of the strength of evidence and supporting the reliability of the review findings.

## 4. Finding

A total of 26 articles were included in the review, revealing several notable trends in AI and XR applications within foodservice settings. The first publications meeting our criteria appeared in 2018, while the majority of the studies (16 out of 26) were published in the last two years of the examined period, indicating a recent surge of interest in the field (see Figure S1). In addition, an examination of the publication outlets reveals a clear disciplinary imbalance, with most studies appearing in engineering/technology journals and comparatively fewer in social science and hospitality-focused journals (see Figure S2). Across the studies, AI-based approaches dominated, particularly in areas such as calorie and nutrition tracking, personalized food recommendations, menu planning, and interventions promoting healthier eating. XR applications, while emerging, were less prevalent but showed promise for immersive, real-time dietary interventions. Overall, the findings indicate a shift from early, static, optimization-focused systems toward adaptive, interactive, and behavior-oriented technological solutions. The following subsections present the reviewed studies in five categories, thereby allowing a more structured discussion and analysis.

### 4.1. AI-Powered Calorie and Nutrition Tracking

Studies in this category reveal a clear progression from isolated image-based dietary assessment toward more integrated and real-time calorie tracking systems. While all studies aim to automate nutritional evaluations, they differ in methodological sophistication, system integration, and applicability across foodservice contexts. The earlier work, epitomized by Siemon et al. [40], uses machine learning with transfer learning and clustering for food segmentation in controlled school foodservices. Similarly situated, Gao et al. [41] present a vision-based dietary assessment system in university canteens. Despite their methodological differences, both rely on static images, thereby limiting real-time application in dynamic restaurant settings. Subsequent studies extended AI-based dietary assessments by combining food recognition and portion estimation into a single framework. Shi et al. [18] integrate food identification, volume estimation, and nutritional analysis for automated tray meal assessment. Building on this work, Shi et al. [42] provide improved calorie estimates through the use of deep learning together with 3D reconstructions. The aforementioned studies exhibit a shift toward more integrated methods that combine sequential dietary assessment steps.

Chang et al. [20], provided an early yet conceptually significant contribution by introducing an AIoT-based system into food buffet environments. By integrating intelligent tables, mobile applications, and cloud platforms, this AIoT approach enables real-time calorie tracking during food selection, signaling representing a shift to in situ decision support and away from post-consumption analyses. Overall, the literature indicates a transition from static, vision-based models to more integrated and adaptive systems. However, most studies confine their focus to institutional settings and prioritize technical accuracy over user interactions, thereby highlighting the need for more context-aware and consumer-facing applications. Table 2 summarizes the studies that focus on AI-powered methods for dietary assessment and calorie tracking.

### 4.2. AI-Driven Personalized Food Recommendations & Meal Guidance

Studies in this category demonstrate a clear evolution from static, preference-based recommendation systems toward adaptive, context-aware, and health-oriented meal guidance. While all approaches aim to support decision-making, they differ in their optimization logic, level of personalization, and ability to influence actual dietary behavior. Early studies primarily focus on preference prediction and constraint-based optimization. Li et al. [10] utilize historical order data and menu evaluations to recommend dishes, particularly in environments with extensive menu options. Similarly, Wang et al. [22] deploy a knowledge-based multi-objective model to balance taste preferences with budget constraints. These systems improve decision efficiency but remain largely static, offering limited responsiveness to changing user needs or health goals, relying heavily on predefined rules and historical patterns. Qiao et al. [43] extend this approach by incorporating nutritional considerations alongside behavioral data, enabling healthier and more personalized dish recommendations with real-time response capabilities and improved privacy protection. Wu et al. [44] further explore this direction through a fully automated diet counseling system that monitors food purchases, demonstrating scalability.

More recent research exhibits a shift toward adaptive and interactive systems. Liu et al. [21] introduce the concept of reinforced learning that enables continuous adaptation evolving user health conditions, marking a transition toward dynamic, real-time recommendation environments. Similarly, Wang et al. [45] enhance personalization by integrating heterogeneous data, while Forouzandeh et al. [46] improve the accuracy of recommendations by modeling semantic relationships between food and health attributes. Vandeputte et al. [23] further highlight the importance of user engagement, showing that interactive systems increase acceptance of healthier alternatives. Overall, the literature reveals a transition from static optimization models to more adaptive, user-centered recommendation systems. Despite the documented progress, challenges remain when translating personalization into sustained behavioral change and integrating contextual, nutritional, and experiential factors. Table 3 summarizes the studies on AI-powered food recommendation systems in restaurant and foodservice settings.

### 4.3. AI-Driven Menu Planning

Research on AI-driven menu planning demonstrates a transition from rule-based nutritional compliance toward dynamic, multi-objective optimization systems that balance health, cost, personalization, and operational efficiency. Early work, such as a paper by Hernandez-Ocana et al. [47], relies on rule-based and bio-inspired algorithms to generate menus aligned with established nutritional guidelines. This approach illustrates the capacity of AI to translate abstract dietary principles into structured meal plans. However, its limited scale and reliance on predefined rules constrain flexibility and real-world adaptabilities. Subsequent studies emphasize optimal performance and scalability. Sahin and Aytekin-Sahin [48] compare advanced multi-objective algorithms and demonstrate that evolutionary optimization methods outperform alternatives by balancing nutritional adequacy with operational constraints. Compared with earlier rule-based systems, these models represent methodological progress by enabling more efficient and scalable menu planning across diverse foodservice settings.

A parallel stream focuses on institutional applications, particularly in schools and college canteens. Studies by Segredo et al. [49] and Marrero et al. [50] show how AI can simultaneously address affordability, nutritional balance, and menu diversity, thereby aligning with public health objectives. Though more context-specific, such systems demonstrate strong practical relevance, especially in controlled environments where requirements are standardized. More recent research introduces user-centered and experiential elements into menu planning. Hannon et al. [51] integrate multimodal AI and user feedback to enable adaptive and personalized menu customization, signaling a shift from system-driven optimization to interactive design processes. In addition, Li et al. [52] extend menu planning from the design stage to execution by incorporating portion recognition in order to reduce food waste and linking planning related decisions to sustainability outcomes. Overall, the literature exhibits progress from static, rule-based models toward adaptive, multi-objective, and increasingly user-informed systems. However, most applications remain concentrated in institutional settings, with limited integration of real-time consumer interactions and the operational complexities which characterize commercial restaurant environments (see Table 4).

### 4.4. AI-Based Promotion of Healthier Eating Choices in Restaurants

This research category reflects a shift from information provision toward behavioral interventions, where food choices are influenced actively by AI systems through persuasion, transparency, and nudging mechanisms. While all studies aim to promote healthier decisions, they differ in the psychological strategies deployed and the level of user engagement. One prominent stream focuses on avatar-based persuasion. Hao et al. [53] show that the effectiveness of avatars depends on design elements such as appearance, humor, and messaging style, which activate social comparisons and aspirational motives. Aman et al. [54] later demonstrate that the embedding of avatars in menu interfaces can both encourage healthier choices and enhance experiential outcomes such as satisfaction and loyalty. When compared with traditional recommendation systems, these approaches emphasize affective and symbolic engagement rather than guidance which is solely functional. A second stream highlights the role of explainability in recommender systems. De Croon et al. [19] show that providing a transparent explanation improves users’ understanding and perceived control, even when recommendations conflict with initial preferences. This suggests that explainability can mitigate resistance and enhance trust, addressing a limitation that is commonplace with opaque AI systems. Explanation-driven systems differ from avatar-based approaches by prioritizing cognitive engagement over emotional influence.

A third approach involves nudging and gamification through decision-support interfaces. Agyemang et al. [55] demonstrate how dashboards can translate abstract nutritional information into actionable insights by incorporating simplified health metrics and environmental feedback. These systems reinforce behaviors through incentives and feedback loops, offering more structured and data-driven interventions. A key distinction emerges across these studies between affective (avatars), cognitive (explanations), and behavioral (nudging) mechanisms. Collectively, the scholarly contributions signal a progression toward multi-dimensional AI systems that influence decision-making by integrating these elements. However, limited evidence exists on their long-term effectiveness and real-world adoption, highlighting the need for further validation in a variety of restaurant settings. Table 5 includes studies that apply AI-based approaches to guide healthier food choices in restaurants through interactive techniques.

### 4.5. Immersive & Real-Time Dietary Interventions

Although AI-based approaches dominated in the literature, XR-based interventions represent a conceptually distinct, albeit smaller stream that emphasizes real-time perception, embodiment, and experiential influence on dietary behavior. A consistent pattern emerges across the three identified studies—XR is not improving nutritional decision-making through an abstract delivery of information. It alters how food is perceived at the moment of choice. Fuchs et al. [29] demonstrate that the integration of Mixed Reality (MR), with Internet of People (IoP) systems, enables continuous dietary feedback in situ thereby embedding behavioral cues directly into the physical environments that are encountered by users. This signals a shift when designing interventions from retrospective monitoring to steering behaviors in real-time. Sharma et al. [28] focus on perceptual influences at the ordering interface, to show that AR menus reduce over-ordering significantly by modifying perceptions of portion size and by placing an increased salience on food waste, thereby influencing the heuristics of pre-consumption decisions. These findings are complemented by Braga et al.’s [56] finding that in VR improves the capacity of users to estimate portion sizes, thereby strengthening cognitive calibrations of serving expectations. Taken together, these studies suggest that rather than relying on algorithmic recommendations XR operates primarily through perceptual distortion correction, immersive cueing, and experiential learning (see Table 6). This positions XR as a layer of behavioral augmentation that complements AI-driven informational systems.

### 4.6. Overall Synthesis of Findings

Across the 26 reviewed studies, a clear distinction emerges between studies on behavioral impacts and others that develop and propose models. A smaller subset of the relevant studies provides validated evidence of impact, primarily in terms of improved nutritional understanding, healthier food choices, or enhanced user engagement (e.g., through interactive recommender systems, explainability features, or XR-based perceptual interventions). These studies typically deploy user experiments, behavioral measurements, or field implementations in foodservice settings. In contrast, a larger proportion of the literature focuses on the development and technical validation of AI models, including food recognition systems, recommendation algorithms, and menu optimization tools. While these studies demonstrate high accuracy and computational performance, they often lack behavioral validation, which limits conclusions about their effectiveness in influencing dietary behaviors. Overall, the findings suggest that the field is transitioning from technically driven systems to more user-centered and behaviorally validated applications. However, robust empirical evidence remains limited, highlighting a gap between technological capabilities and their demonstrated impact on consumer behavior.

### 4.7. Other Related Studies

Much research has targeted individuals rather than restaurants or other foodservice establishments and has shown the importance of providing personalized menus and nutritional recommendations tailored to customers. This area of research is broad and requires a focused systematic literature review to allow for deeper exploration, divided into two main categories:

*Personalized meal plans—Individual Focus.* The first category primarily targets end consumers seeking personalized meal plans to support a healthy lifestyle (e.g., [57,58]). In these studies, researchers typically propose apps or systems designed for individuals with specific dietary needs, ranging from generally healthy individuals to those managing particular health conditions. 

*Food recognition and dietary assessment systems.* The second category includes studies that have developed deep learning-based food recognition and dietary assessment systems to analyze daily meal images (e.g., [17,59,60,61,62,63,64,65]). These systems are designed for individuals seeking personalized nutritional insights to improve health and wellness and typically employ advanced algorithms to identify food items and assess their nutritional content. Although most of these studies focus on individual users, their underlying technologies have potential applications in restaurants and other foodservice contexts. 

*AI and XR Applications for Enhancing Nutritional Management in Healthcare industry.* Healthcare represents a distinct and mature research domain in which AI and XR have been extensively applied to nutritional management across hospitals, clinics, elder care, and digital health platforms. Prior studies demonstrate how AI systems support clinical nutrition through automated intake estimation and disease-specific dietary control. For example, Lu et al. [66] developed an AI-based system to estimate hospitalized patients’ nutrient intake, while Jin et al. [67] examined AI-driven dietary recommendations for hemodialysis patients to manage potassium levels. The breadth and clinical specificity of this literature distinguish it from foodservice-focused research, warranting separate systematic review treatment.

## 5. Discussion

### 5.1. Conceptual Framework Linking AI/XR, Nutrition Awareness, and Health-Conscious Choices

The findings have been organized using an integrated conceptual framework that combines the Technology Acceptance Model (TAM) and the Health Belief Model (HBM). TAM has been widely employed to explain consumer adoption and use of technology-based systems emphasizing perceived usefulness and perceived ease of use as key determinants of engagement with technological interfaces (e.g., [68]). In contrast, HBM has been extensively applied to understand health-related decision-making, focusing on perceived benefits, perceived barriers, and cues to action that influence behavioral change (e.g., [69]).

In the context of restaurants and foodservice operations, AI- and XR-enabled applications (e.g., intelligent menus, recommender systems, and immersive visualizations) serve as technological stimuli that consumers must first accept and interact with. AI technologies, such as recommendation engines and chatbots, provide personalized nutrition guidance, (e.g., [10]) while XR tools, including AR menus and VR simulations, offer immersive, interactive experiences (e.g., [56]). Together, they complement each other by enhancing awareness and promoting healthier consumer food choices [29]. When these technologies are perceived as useful and easy to use, they enhance consumers’ nutrition awareness by improving understanding of calorie content, portion sizes, ingredient composition, and dietary implications [11,28,34,68]. This heightened awareness subsequently activates health-related beliefs by clarifying the benefits of healthier choices and providing salient cues to action at the point of decision-making, ultimately supporting more health-conscious food and beverage selections.

Accordingly, the reviewed studies can be conceptually positioned along a sequential pathway linking (1) AI and XR technological characteristics, (2) consumer nutrition awareness, and (3) health-conscious decision-making outcomes. This framework provides a unifying lens for synthesizing the heterogeneous literature reviewed in this study and clarifies how technological design, consumer cognition, and behavioral responses are interconnected in foodservice settings.

**Figure 3:**
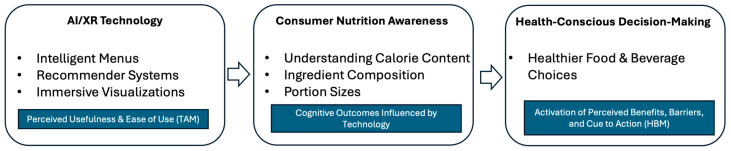
Conceptual Framework: The Role of AI/XR in Enhancing Nutrition Awareness and Health-Conscious Choices.

Figure 3 illustrates the conceptual framework, showing how AI/XR technology influences nutrition awareness and health-conscious choices. The model highlights the evolution from focusing solely on technology adoption (TAM) to integrating psychological and behavioral mechanisms (HBM) that guide food decisions. By linking technological characteristics to cognitive awareness and health-related beliefs, the framework emphasizes both the informational and motivational roles of AI/XR in promoting healthier consumer choices in foodservice settings.

### 5.2. Trends in AI and XR Research over Time (2016–2025)

Over the past decade, research on AI and XR in foodservice settings has evolved through several phases. Early studies (2016–2019) focused primarily on static, optimization-based approaches, such as rule-based menu planning and preference-driven recommendation systems. Between 2020 and 2021, the literature expanded into institutional contexts, incorporating deep learning, reinforcement learning, and AIoT to address challenges related to cost, dietary diversity, and calorie control. From 2022 onward, a clear shift was evident toward real-time, adaptive, and user-centric systems, characterized by the integration of computer vision, natural language processing, and multimodal AI. More recent (2024–2025) studies are reflective of a transition toward behavior-oriented and decision-support ecosystems, emphasizing explainability, gamification, avatars, and automated dietary assessment.

This progression indicates a broader paradigm shift from purely technical optimization toward intelligent, interactive systems designed to influence consumer behavior and promote healthier and more sustainable food choices. At the same time, XR-based applications remain comparatively underexplored, highlighting an imbalance in the technological development trajectory and reinforcing the need for further research on immersive interventions. Furthermore, the concentration of studies in the most recent years indicates that the field remains in a relatively early stage of development. While technological innovation is accelerating, the limited theoretical integration highlights the need for more robust and theory-driven research to support sustainable adoption in real-world foodservice environments.

### 5.3. Future Research Directions

In addition to the three main relevant areas (personalized meal plans, food recognition systems, and a focus on the healthcare industry) that require separate systematic literature reviews, this study has identified several other potential research directions. Research on AI and XR in this domain follows two main perspectives. The first emphasizes improving the technical accuracy of AI and XR for tasks such as food recommendation and detection, requiring substantial technical expertise and receiving extensive attention in nutrition and computer science research (e.g., [49,56]). The second, prevalent in hospitality management, explores how these technologies support informed food and beverage choices (e.g., [53,54]). While the technical stream is well developed, social science research, particularly in hospitality, remains limited, highlighting opportunities to integrate consumer behavior and service theories into AI and XR design and evaluation. Accordingly, future research should examine whether AI- and XR-enabled menu systems designed using hospitality and consumer behavior theories lead to higher customer trust, satisfaction, and adoption intentions compared to systems developed without such theoretical grounding.

Also observed from the results, while numerous studies have been conducted utilizing AI, there is a noticeable lack of research exploring the use of Extended Reality technologies. While AI has garnered significant attention for its applications, XR has received comparatively less. There remains a considerable opportunity for future researchers to investigate the potential of XR in this area, especially in applications where immersive experiences could offer innovative solutions. Future studies should investigate how XR-based menu interventions (e.g., AR and VR) differ from AI-only systems in influencing nutrition awareness, portion perception, and ordering behavior in foodservice settings.

It has also been observed that much research has focused on food rather than on beverage, particularly in the case of liquor. Alcoholic beverages represent a significant component of consumer demand and spending in many markets [70], yet they remain underexplored by researchers. Conducting studies on beverages in general, and liquors in particular, could help individuals have a better and more informed experience in the hospitality industry. There is a need for further research to explore how AI- and XR-enabled menu and recommendation systems for beverages, particularly alcoholic drinks, influence consumer awareness, moderation intentions, and beverage selection in hospitality contexts.

Furthermore, there has been limited scholarly exploration regarding customer perceptions of these interventions. Human–food interactions play a significant role in the overall enjoyment of life [71]. Many individuals prefer to disconnect from technology while eating [72], valuing the opportunity to be in a cozy environment, listen to pleasant music [73], enjoy conversations with those at the dining table, and savor their food without tech-related distractions. Future researchers could explore how to balance technological interventions with the desire for more authentic, technology-free dining experiences. This highlights the need for future research to examine how customers perceive and respond to AI- and XR-enabled foodservice interventions when dining authenticity and technology avoidance motivations are salient.

An important yet underexplored issue in foodservice settings is meeting individual nutritional needs. A central goal in this field is supporting consistency in achieving essential nutritional requirements [74]. However, many diners make food choices with limited understanding of their nutritional needs, relying heavily on guesswork, which highlights a gap in personalized nutrition tracking. Future research could focus on developing comprehensive databases that monitor individuals’ nutritional intake over defined periods (e.g., weekly or monthly). Such systems could function as personalized portfolios, offering insights into dietary patterns and enabling more informed meal planning. These databases could also store preferences, allergies, budget constraints, health conditions, and related factors. If restaurants contributed standardized nutritional data, customer profiles could be scanned across establishments to deliver tailored menu options. Recommendations could then be customized based on taste preferences, nutritional needs, price sensitivity, and variety, enhancing personalization and supporting more informed dining decisions. Future researchers may also explore whether access to longitudinal, cross-restaurant personalized nutrition profiles enhances consumer trust in AI-driven menu recommendations and improves alignment between menu selections and individual nutritional needs.

Beyond identifying technological and contextual gaps, future research should examine the psychological and behavioral mechanisms through which AI and XR influence healthier food choices. Studies should empirically test cognitive load reduction as a mediator in AI-based menu systems and its effect on food selection in complex dining environments. Similarly, research should investigate immersion as a mediating mechanism in XR-based menus, focusing on how visual realism and interactivity shape portion perception, ordering behavior, and consumption intentions. Trust, transparency, and explainability should also be examined as mediators between AI-driven recommendations and consumer acceptance, particularly across culturally diverse foodservice contexts. Modeling these mechanisms would advance theory beyond adoption outcomes and clarify how AI and XR operate as behavioral interventions in restaurant settings, and future studies should further investigate the psychological mechanisms (e.g., cognitive load reduction, immersion, trust, and explainability) that mediate the relationship between AI/XR interventions and healthier food choices in restaurant environments.

### 5.4. Practical Implications

The complexity and profusion of choices presented in modern menus can overwhelm consumers, undermining meal selections that align with personal preferences and dietary requirements. This decision fatigue is particularly pronounced in foodservice settings where menu options are diverse and frequently include complex ingredient lists. To mitigate this challenge, restaurants and foodservice providers can leverage AI and XR-powered menus to introduce a more intuitive and tailored dining experience. By integrating an advanced search engine within the menu interface, restaurants can enable customers to filter meal options based on customizable criteria such as dietary preferences (e.g., vegetarian, gluten-free), nutritional goals (e.g., low-calorie, low-sodium), or specific ingredients (e.g., including rice, excluding dairy). This feature would empower customers to make more informed and efficient meal choices, enhancing their overall dining experience.

In addition to improving the customer experience, AI and XR-driven menu systems can also foster deeper engagement by offering personalized meal recommendations that adapt to customer preferences over time. As customers return, the system could refine its suggestions based on previous selections, dietary restrictions, and even real-time health data (if applicable), thus creating a more dynamic and personalized dining journey. For foodservice providers, implementing such technologies can increase customer satisfaction, reduce decision fatigue, and enhance operational efficiency. By streamlining the decision-making process, restaurants can enhance service delivery, improve order accuracy, and boost customer loyalty. Moreover, AI-driven menu systems could generate valuable data insights, enabling restaurants to tailor their offerings to meet changing customer demands and emerging dietary trends.

However, alongside these benefits, the implementation of AI- and XR-enabled menu systems introduces important operational and legal responsibilities for foodservice providers. As these technologies increasingly influence consumers’ dietary decisions, restaurants must ensure the accuracy and reliability of nutritional information, particularly in relation to allergens and dietary restrictions. Inaccurate or incomplete recommendations may not only harm consumers but also expose providers to potential liability and undermine customer trust. To mitigate these risks, restaurants should incorporate clear disclaimers, maintain up-to-date ingredient and nutritional databases, and design systems that prioritize transparency and user verification. Additionally, close collaboration with technology developers is essential to ensure that AI-driven recommendations comply with food safety regulations and industry standards, thereby supporting both consumer protection and responsible innovation.

### 5.5. Limitations

Despite its methodological rigor, interdisciplinary scope, and strong theoretical foundation, this study has several limitations that should be acknowledged. First, the literature search was conducted primarily in English, potentially excluding relevant studies published in other languages, which may limit the global comprehensiveness of the findings. Second, since the study is based on qualitative content analysis, its conclusions should be interpreted with caution regarding generalizability. Third, the review relies on published journal articles, and excludes conference papers, research notes, and grey literature, which may have resulted in the omission of emerging or innovative AI and XR applications. Finally, this review covers a decade of research, from 2016 to 2025, which may result in a lengthy overview and could potentially miss the very latest developments beyond this period.

## 6. Conclusions

The integration of AI and XR technologies within the food and beverage sector offers significant benefits for a variety of stakeholders. For consumers, these technologies enable more informed meal choices by providing real-time insights into portion sizes, nutritional content, and personal preferences, thereby promoting healthier eating behaviors. For foodservice providers, AI and XR tools can enhance operational efficiency, improve customer satisfaction, and contribute to a more tailored dining experience. This personalized approach optimizes service delivery, fosters customer loyalty, and supports broader societal goals by encouraging better eating habits. However, adopting these technologies involves challenges, including varying consumer acceptance, operational costs, integration with existing workflows, and ensuring that digital interventions support rather than overshadow the social and experiential aspects of dining. Future researchers may explore the role of XR technologies in foodservice, and particularly their potential to create immersive and behavior-influencing dining experiences. Greater attention should also be given to consumer perceptions, beverage-related applications, and the development of personalized, longitudinal nutrition systems. Additionally, integrating behavioral and hospitality theories into AI and XR design remains essential to improving user trust, adoption, and overall effectiveness, ultimately enabling these technologies to reach their full potential in supporting healthier and more engaging dining experiences.

## Figures and Tables

**Table 1:** Eligibility Criteria for Study Selection.

Inclusion Criteria	Exclusion Criteria
Published between 2016 and 2025	Studies not available in full text
Peer-reviewed articles	Non-peer-reviewed articles
Written in English	Reviewed papers
Empirical research articles	Studies related to personal use rather than foodservice
Studies on AI or XR in menus and nutritional labeling in restaurants/foodservice settings	Healthcare related studies
	Animal nutrition studies

**Table 2:** AI-powered calorie and nutrition tracking. Sources are listed in chronological order by year of publication.

Reference(s)	Journal	Targeted Foodservice in the Study	Mapped AI Category	Key Contribution/Finding
[40]	Scientific Reports	School foodservices	Machine Learning/Deep Learning	Proposed a novel approach combining transfer learning and clustering for improved food segmentation
[20]	EEE Transactions on Consumer Electronics	Buffet restaurants	Machine Learning/Deep Learning/Reinforcement Learning	Introduced a AIoT-based intelligent system for buffet meal calorie control
[18]	Food chemistry	Restaurants	Computer Vision/Machine Learning	Developed the pioneering dataset for Chinese tray meals and proposed a novel system for automated dietary evaluation of tray meals.
[41]	Food Chemistry	University canteens	Computer Vision	Introduced a vision-based system for dietary assessment of college students
[42]	Computers and Electronics in Agriculture	Foodservice providers	Computer Vision/Deep Learning	Integrates AI with 3D reconstruction for calorie detection

**Table 3:** Personalized food recommendations & meal guidance. Sources are listed in chronological order by year of publication.

Reference(s)	Journal	Targeted Foodservice in the Study	Mapped AI Category	Key Contribution/Finding
[10]	Frontiers in psychiatry	Restaurants, foodservice chains, fast food chains	Recommender Systems	Development of relation-driven food recommendation system predicting dietary preferences (ingredients, spice, price, nutrition).
[22]	Applied Soft Computing	Restaurants, foodservice operators	Knowledge-Based Systems	knowledge-based multi-objective optimization algorithm for suggesting food packages
[43]	Transactions on Emerging Telecommunications Technologies	Restaurants, catering services	Machine Learning/Data Mining	Provided healthier, personalized dish recommendations with real-time performance and improved privacy protection.
[23]	The Journal of Nutrition	Foodservices	Data Mining/Recommender Systems	Higher acceptance of food swaps when users engaged
[46]	Computers in Biology and Medicine	Restaurants	Machine Learning	Enhances unsupervised learning of attributed graph representations, outperforms existing methods.
[21]	Expert Systems with Applications	Restaurants	Reinforcement Learning	Proposed dynamic, adaptive FRS for real-time recommendations.
[45]	Egyptian Informatics Journal	Restaurants	Machine Learning	Enables more dynamic and personalized recommendations.
[44]	IEEE Journal of Biomedical and Health Informatics	Foodservice	Machine Learning/Recommender Systems	Fully automated diet counseling using digital receipts and structured recommendations

**Table 4:** Meal & menu planning systems. Sources are listed in chronological order by year of publication.

Reference(s)	Article No and Journal	Targeted Foodservice in the Study	Mapped AI Category	Key Contribution/Finding
[47]	IEEE Access	Foodservices	Machine Learning	Adapted BFOA to generate personalized menus satisfying nutritional needs according to “Laws of Nutrition”
[49]	IEEE access	School cafeterias	Machine Learning	Developed an AI-driven menu planner for school canteens that balances energy, nutrition, affordability, and diversity
[50]	Mathematics	School foodservices	Machine Learning	Addressed repetition and cost in school menu planning
[51]	Computers	Restaurants	Natural Language Processing (NLP)/Computer Vision	Uses multimodal AI to offer personalized recipe recommendations based on dietary restrictions and preferences
[48]	Expert Systems with Applications	Restaurants, Schools, Catering Services	Knowledge-Based Systems	First use of AGEMOEA and SMSEMOA for menu planning; developed open-source tool EvoMeal
[52]	Applied Sciences	College foodservice	Computer Vision/Deep Learning	Automatic plate recognition system for food waste reduction

**Table 5:** AI-based Promote Healthier Eating Choices in Restaurants. Sources are listed in chronological order by year of publication.

Reference(s)	Journal	Targeted Foodservice in the Study	Mapped AI Category	Key Contribution/Finding
[55]	Science of the Total Environment	Restaurants, Campus cafeterias	Recommender Systems/Reinforcement Learning	Using a dashboard and gamification to promote healthier, more environmentally friendly food choices
[53]	International Journal of Contemporary Hospitality Management	Restaurants, health-focused food services	Natural Language Processing (NLP)/Computer Visio	Focused on avatar appearance, playfulness, and persuasive messaging to promote healthier choices
[19]	JMIR mHealth and uHealth	Foodservice	Deep Learning/Natural Language Processing (NLP)/Recommender Systems	Increased user understanding, control, and healthier decisions by Integrating explanations to guide food choices
[54]	Tourism Review	Restaurants	Natural Language Processing (NLP)/Computer Vision	Use of Avatars as a Digital Nudge

**Table 6:** Immersive & real-time dietary interventions. Sources are listed in chronological order by year of publication.

Reference(s)	Journal	Targeted Foodservice in the Study	Mapped XR/AI Category	Key Contribution/Finding
[29]	Future Generation Computer Systems	Restaurants, Smart vending, cafeterias	Mixed Reality (MR)/Computer Vision	MR-based system for real-time diet-related interventions using head-mounted cameras; improved beverage choices (less sugar/energy) and healthier food choices (less saturated fat)
[28]	Current Issues in Tourism	Restaurants, menu designers, AR developers	Augmented Reality (AR)	Mitigated over-ordering by altering portion size perceptions and increasing awareness of food waste by using AR menus instead of picture-based menus
[56]	Journal of Nutrition Education and Behavior	Restaurants,	Virtual Reality (VR)	Improved portion control self-efficacy and portion-size estimation accuracy

## Data Availability

No new data were created or analyzed in this study.

## Supplementary Materials

The following supporting information can be downloaded at: https://www.mdpi.com/article/10.3390/nu18091364/s1, Table S1: Quality assessment; Figure S1: Year and the number of publications; Figure S2: Distribution of selected articles in engineering/technology journals and social science/hospitality journals.

## References

[1] Reiners D., Davahli M.R., Karwowski W., Cruz-Neira C. (2021). The combination of artificial intelligence and extended reality: A systematic review. Front. Virtual Real..

[2] Chen S., López-Gil J.F., Memon A.R., Bao R., Yang X. (2024). Associations of eating habits with self-rated health and life satisfaction in adolescents: A 42-country cross-sectional study. Eur. J. Investig. Health Psychol. Educ..

[3] Woodward J., Ruiz J. (2022). Analytic review of using augmented reality for situational awareness. IEEE Trans. Vis. Comput. Graph..

[4] Samant S., Bakhos J.J., Wu W., Zhao S., Kassab G.S., Khan B., Panagopoulos A., Makadia J., Oguz U.M., Banga A. (2023). Artificial intelligence, computational simulations, and extended reality in cardiovascular interventions. JACC Cardiovasc. Interv..

[5] Alimamy S., Gnoth J. (2022). I want it my way! The effect of perceptions of personalization through augmented reality and online shopping on customer intentions to co-create value. Comput. Hum. Behav..

[6] Mayer A., Greif L., Häußermann T.M., Otto S., Kastner K., El Bobbou S., Chardonnet J.R., Reichwald J., Fleischer J., Ovtcharova J. (2025). Digital Twins, Extended Reality, and Artificial Intelligence in Manufacturing Reconfiguration: A Systematic Literature Review. Sustainability.

[7] Mun S.G., Woo L., Paek S. (2019). How important is F&B operation in the hotel industry? Empirical evidence in the US market. Tour. Manag..

[8] Namkung Y., Jang S. (2007). Does food quality really matter in restaurants? Its impact on customer satisfaction and behavioral intentions. J. Hosp. Tour. Res..

[9] Abbas R., Hatch C.D. (2024). Providing Nutrition Information on US Restaurant Menus: A Systematic Review Since the Affordable Care Act (2010). J. Consum. Policy.

[10] Li X., Jia W., Yang Z., Li Y., Yuan D., Zhang H., Sun M. (2018). Application of intelligent recommendation techniques for consumers’ food choices in restaurants. Front. Psychiatry.

[11] Davis F.D. (1985). A Technology Acceptance Model for Empirically Testing New End-User Information Systems: Theory and Results. Ph.D. Thesis.

[12] Becker M.H. (1974). The health belief model and personal health behavior. Health Educ. Monogr..

[13] Van Dyke N., Drinkwater E.J. (2014). Review article relationships between intuitive eating and health indicators: Literature review. Public Health Nutr..

[14] Nie X., Zhao W.M., Guan J. (2025). Towards a framework of menu research: Insights from the multiple level perspective and signaling theory. Br. Food J..

[15] Hassannejad H., Matrella G., Ciampolini P., De Munari I., Mordonini M., Cagnoni S. (2017). Automatic diet monitoring: A review of computer vision and wearable sensor-based methods. Int. J. Food Sci. Nutr..

[16] Bo X.I.A., Abidin M.R.Z., Ab Karim S. (2024). From tradition to technology: A comprehensive review of contemporary food design. Int. J. Gastron. Food Sci..

[17] Herranz L., Jiang S., Xu R. (2016). Modeling restaurant context for food recognition. IEEE Trans. Multimed..

[18] Shi J., Han Q., Cao Z., Wang Z. (2024). DeepTrayMeal: Automatic dietary assessment for Chinese tray meals based on deep learning. Food Chem..

[19] De Croon R., Segovia-Lizano D., Finglas P., Vanden Abeele V., Verbert K. (2025). An explanation interface for healthy food recommendations in a real-life workplace deployment: User-centered design study. JMIR Mhealth Uhealth.

[20] Chang W.J., Chen L.B., Lin I.C., Ou Y.K. (2021). iBuffet: An AIoT-based intelligent calorie management system for eating buffet meals with calorie intake control. IEEE Trans. Consum. Electron..

[21] Liu L., Guan Y., Wang Z., Shen R., Zheng G., Fu X., Yu X., Jiang J. (2024). An interactive food recommendation system using reinforcement learning. Expert Syst. Appl..

[22] Wang Z., Meng C., Ji S., Li T., Zheng Y. (2020). Food package suggestion system based on multi-objective optimization: A case study on a real-world restaurant. Appl. Soft Comput..

[23] Vandeputte J., Herold P., Kuslii M., Viappiani P., Muller L., Martin C., Davidenko O., Delaere F., Manfredotti C., Cornuéjols A. (2023). Principles and validations of an artificial intelligence-based recommender system suggesting acceptable food changes. J. Nutr..

[24] Trang Tran T.N., Atas M., Felfernig A., Stettinger M. (2018). An overview of recommender systems in the healthy food domain. J. Intell. Inf. Syst..

[25] Abhari S., Safdari R., Azadbakht L., Lankarani K.B., Kalhori S.R.N., Honarvar B., Abhari K., Ayyoubzadeh S.M., Karbasi Z., Zakerabasali S. (2019). A systematic review of nutrition recommendation systems: With focus on technical aspects. J. Biomed. Phys. Eng..

[26] Saad A.M., Islam M.M. (2025). Navigating nutrients: A scoping review on real-time food nutrition classification and recommendation systems. Comput. Biol. Med..

[27] Persky S., Dolwick A.P. (2020). Olfactory perception and presence in a virtual reality food environment. Front. Virtual Real..

[28] Sharma S., Singh G., Prasad B. (2024). Portion perception and waste awareness: Investigating the effects of Augmented Reality menus on over-ordering intentions in various dining settings. Curr. Issues Tour..

[29] Fuchs K., Haldimann M., Grundmann T., Fleisch E. (2020). Supporting food choices in the Internet of People: Automatic detection of diet-related activities and display of real-time interventions via mixed reality headsets. Future Gener. Comput. Syst..

[30] Deng D.S., Seo S., Harrington R.J., Martin D. (2024). When virtual others are with me: Exploring the influence of social presence in virtual reality wine tourism experiences. Int. J. Wine Bus. Res..

[31] Fritz W., Hadi R., Stephen A. (2023). From tablet to table: How augmented reality influences food desirability. Acad. Mark. Sci..

[32] Sung E.C., Bae S., Han D.I.D., Kwon O. (2021). Consumer engagement via interactive artificial intelligence and mixed reality. Int. J. Inf. Manag..

[33] tom Dieck M.C., Han D.I.D., Rauschnabel P.A. (2024). Augmented reality marketing in hospitality and tourism: A guide for researchers and managers. Int. J. Contemp. Hosp. Manag..

[34] ChanLin L.J., Chan K.C., Wang C.R. (2019). An epistemological assessment of learning nutritional information with augmented reality. Electron. Libr..

[35] Chark R., Ip M.M.H. (2023). Is menu design effective? A p-curving analysis. Int. J. Hosp. Manag..

[36] Moher D., Liberati A., Tetzlaff J., Altman D.G., Prisma Group (2010). Preferred reporting items for systematic reviews and meta-analyses: The PRISMA statement. Int. J. Surg..

[37] Macdonald M., Misener R.M., Weeks L., Helwig M. (2016). Covidence vs Excel for the title and abstract review stage of a systematic review. JBI Evid. Implement..

[38] McHugh M.L. (2012). Interrater reliability: The kappa statistic. Biochem. Med..

[39] Hawker S., Payne S., Kerr C., Hardey M., Powell J. (2002). Appraising the evidence: Reviewing disparate data systematically. Qual. Health Res..

[40] Siemon M.S., Shihavuddin A.S.M., Ravn-Haren G. (2021). Sequential transfer learning based on hierarchical clustering for improved performance in deep learning based food segmentation. Sci. Rep..

[41] Gao Z., Yuan X., Lei J., Guo H., Marinello F., Guerrini L., Carraro A. (2025). A vision-based dietary survey and assessment system for college students in China. Food Chem..

[42] Shi Y., Gao W., Shen T., Li W., Li Z., Huang X., Li C., Chen H., Zou X., Shi J. (2025). Calorie detection in dishes based on deep learning and 3D reconstruction. Comput. Electron. Agric..

[43] Qiao Y., Sun Q., Cao H., Wang J., Hao T. (2022). Privacy-preserving dish-recommendation for food nutrition through edging computing. Trans. Emerg. Telecommun. Technol..

[44] Wu J., Mayer S., Pilz S., Antille Y.S., Albert J.L., Stoll M., Garcia K., Fuchs K., Bally L., Eichelberger L. (2025). FoodCoach: Fully Automated Diet Counseling. IEEE J. Biomed. Health Inform..

[45] Wang J., Zhou J., Aksoy M., Sharma N., Rahman M.A., Zain J.M., Alenazi M.J.F., Aminzadeh A. (2024). Improving healthy food recommender systems through heterogeneous hypergraph learning. Egypt. Inform. J..

[46] Forouzandeh S., Rostami M., Berahmand K., Sheikhpour R. (2024). Health-aware food recommendation system with dual attention in heterogeneous graphs. Comput. Biol. Med..

[47] Hernandez-Ocana B., Chavez-Bosquez O., Hernandez-Torruco J., Canul-Reich J., Pozos-Parra P. (2018). Bacterial foraging optimization algorithm for menu planning. IEEE Access.

[48] Sahin O., Aytekin-Sahin G. (2024). Open-source multi-objective optimization software for menu planning. Expert Syst. Appl..

[49] Segredo E., Miranda G., Ramos J.M., León C., Rodriguez-Leon C. (2020). Schoolthy: Automatic menu planner for healthy and balanced school meals. IEEE Access.

[50] Marrero A., Segredo E., León C., Segura C. (2020). A memetic decomposition-based multi-objective evolutionary algorithm applied to a constrained menu planning problem. J. Math..

[51] Hannon B., Kumar Y., Li J.J., Morreale P. (2024). Chef Dalle: Transforming cooking with multi-model multimodal AI. Computers.

[52] Li Y., Zhang C., Xu H., Yang Y., Lu H., Deng L. (2025). Strategies for Automated Identification of Food Waste in University Cafeterias: A Machine Vision Recognition Approach. Appl. Sci..

[53] Hao F., Aman A.M., Zhang C. (2024). What is beautiful is good: Attractive avatars for healthier dining and satisfaction. Int. J. Contemp. Hosp. Manag..

[54] Aman A.M., Ng W., Hao F., Zhang C., Chon K.K.S. (2025). Digital nudge persuasiveness of avatars in restaurants toward healthy choices and happy diners. Tour. Rev..

[55] Agyemang P., Kwofie E.M., Baum J.I., Wang D. (2024). The design and development of a dashboard for improving sustainable healthy food choices. Sci. Total Environ..

[56] Braga B.C., Long J., Maksi S., Sajjadi P.K., Klippel A., Masterson T.D. (2025). Immersive Virtual Reality Dietitian Improves Portion Control Self-Efficacy and Portion Size Estimation Accuracy. J. Nutr. Educ. Behav..

[57] Kopitar L., Bedrač L., Strath L.J., Bian J., Stiglic G. (2025). Improving Personalized Meal Planning with Large Language Models: Identifying and Decomposing Compound Ingredients. Nutrients.

[58] Stefanidis K., Tsatsou D., Konstantinidis D., Gymnopoulos L., Daras P., Wilson-Barnes S., Hart K., Cornelissen V., Decorte E., Lalama E. (2022). PROTEIN AI advisor: A knowledge-based recommendation framework using expert-validated meals for healthy diets. Nutrients.

[59] Chen X., Johnson E., Kulkarni A., Ding C., Ranelli N., Chen Y., Xu R. (2021). An exploratory approach to deriving nutrition information of restaurant food from crowdsourced food images: Case of Hartford. Nutrients.

[60] Larke J.A., Chin E.L., Bouzid Y.Y., Nguyen T., Vainberg Y., Lee D.H., Pirsiavash H., Smilowitz J.T., Lemay D.G. (2023). Surveying nutrient assessment with photographs of meals (SNAPMe): A benchmark dataset of food photos for dietary assessment. Nutrients.

[61] Li X., Yin A., Choi H.Y., Chan V., Allman-Farinelli M., Chen J. (2024). Evaluating the quality and comparative validity of manual food logging and artificial intelligence-enabled food image recognition in apps for nutrition care. Nutrients.

[62] Lyu W., Seok N., Chen X., Xu R. (2023). Using Crowdsourced Food Image Data for Assessing Restaurant Nutrition Environment: A Validation Study. Nutrients.

[63] O’Hara C., Kent G., Flynn A.C., Gibney E.R., Timon C.M. (2025). An evaluation of ChatGPT for nutrient content estimation from meal photographs. Nutrients.

[64] Papathanail I., Abdur Rahman L., Brigato L., Bez N.S., Vasiloglou M.F., van der Horst K., Mougiakakou S. (2023). The nutritional content of meal images in free-living conditions—Automatic assessment with gofoodtm. Nutrients.

[65] Vasiloglou M.F., Lu Y., Stathopoulou T., Papathanail I., Faeh D., Ghosh A., Baumann M., Mougiakakou S. (2020). Assessing mediterranean diet adherence with the smartphone: The medipiatto project. Nutrients.

[66] Lu Y., Stathopoulou T., Vasiloglou M.F., Christodoulidis S., Stanga Z., Mougiakakou S. (2020). An Artificial Intelligence-Based System to Assess Nutrient Intake for Hospitalised Patients. IEEE Trans. Multimed..

[67] Jin H., Lin Q., Lu J., Hu C., Lu B., Jiang N., Wu S., Li X. (2024). Evaluating the effectiveness of a generative pretrained transformer-based dietary recommendation system in managing potassium intake for hemodialysis patients. J. Ren. Nutr..

[68] Oyman M., Bal D., Ozer S. (2022). Extending the technology acceptance model to explain how perceived augmented reality affects consumers’ perceptions. Comput. Hum. Behav..

[69] Glick A.A., Winham D.M., Heer M.M., Shelley M.C., Hutchins A.M. (2024). Health belief model predicts likelihood of eating nutrient-rich foods among US adults. Nutrients.

[70] Fogarty J. (2010). The demand for beer, wine and spirits: A survey of the literature. J. Econ. Surv..

[71] Batat W., Peter P.C., Moscato E.M., Castro I.A., Chan S., Chugani S., Muldrow A. (2019). The experiential pleasure of food: A savoring journey to food well-being. J. Bus. Res..

[72] Tabares-Tabares M., Aznar L.A.M., Aguilera-Cervantes V.G., León-Landa E., López-Espinoza A. (2022). Screen use during food consumption: Does it cause increased food intake? A systematic review. Appetite.

[73] Ryu K., Lee H.R., Gon Kim W. (2012). The influence of the quality of the physical environment, food, and service on restaurant image, customer perceived value, customer satisfaction, and behavioral intentions. Int. J. Contemp. Hosp. Manag..

[74] Meyers L.D., Hellwig J.P., Otten J.J., Meyers L.D., Hellwig J.P., Otten J.J. (2006). Dietary reference intakes: The essential guide to nutrient requirements. Dietary Reference Intakes.

